# Supplementary material for: Phosphorylation Protects Oncogenic RAS from LZTR1-Mediated Degradation
Source: bioRxiv. 2026 Jan 7:2026.01.07.698128. Preprint. [Version 1] doi: 10.64898/2026.01.07.698128 (PMC12803243; doi:10.64898/2026.01.07.698128)
Supplement: Supplement 1 [file media-1.pdf]

Supplementary Figures and Legends

Figure S1

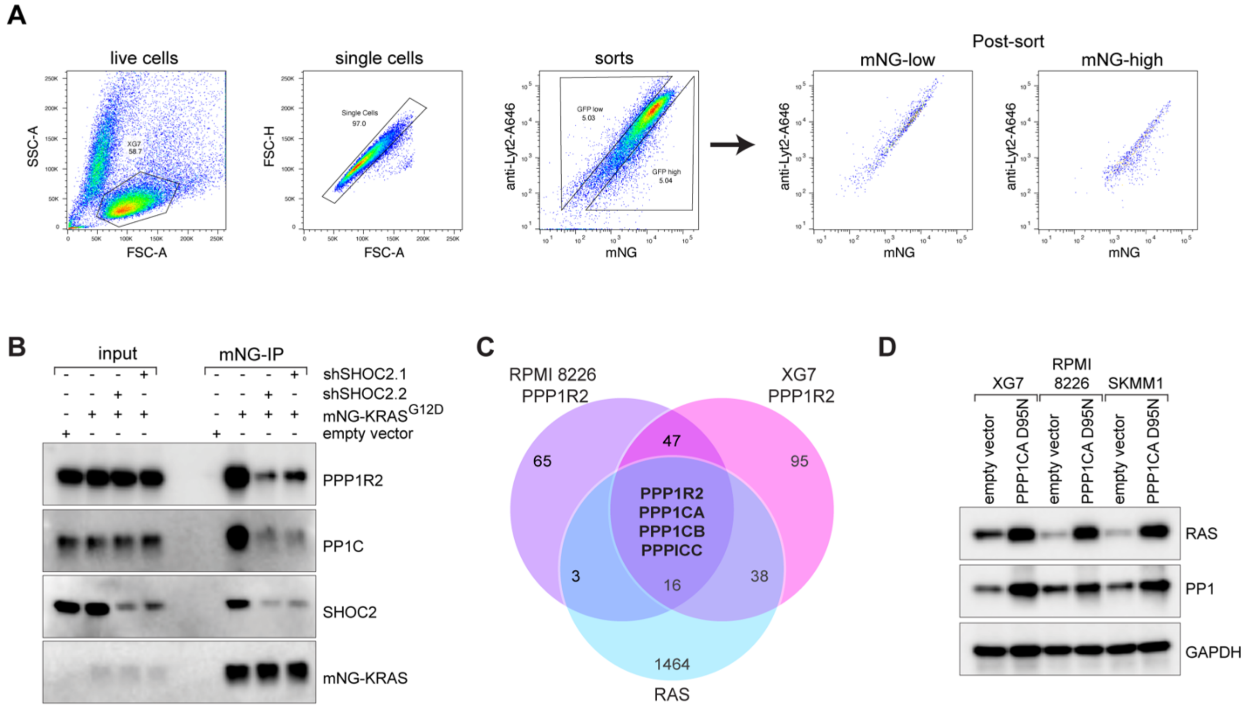

Supplementary Figure 1. PPP1R2 and PP1C regulate RAS stability. A) FACS sorting workflow for mNG-KRAS-low and -high cells. **B)** Co-IP with western blot analysis of $KRAS^{G12D}$ pulldown with PPP1R2, PPP1C and SHOC2 following transduction with shCTRL or two SHOC2 shRNAs, n=2. **C)** Venn diagram of overlapping genes from RAS BioID2 proteomics (≥1.0 log2 enrichment vs. control (*5*)), PPP1R2 BioID2 proteomics (≥1.0 log2 enrichment vs. control) in XG7 and RPMI 8226 cells. **D)** Immunoblot analysis of RAS, PP1C and GAPDH after transduction with empty vector or DN PP1C in XG7, RPMI 8226 and SKMM1 cells, n=4.

Figure S2

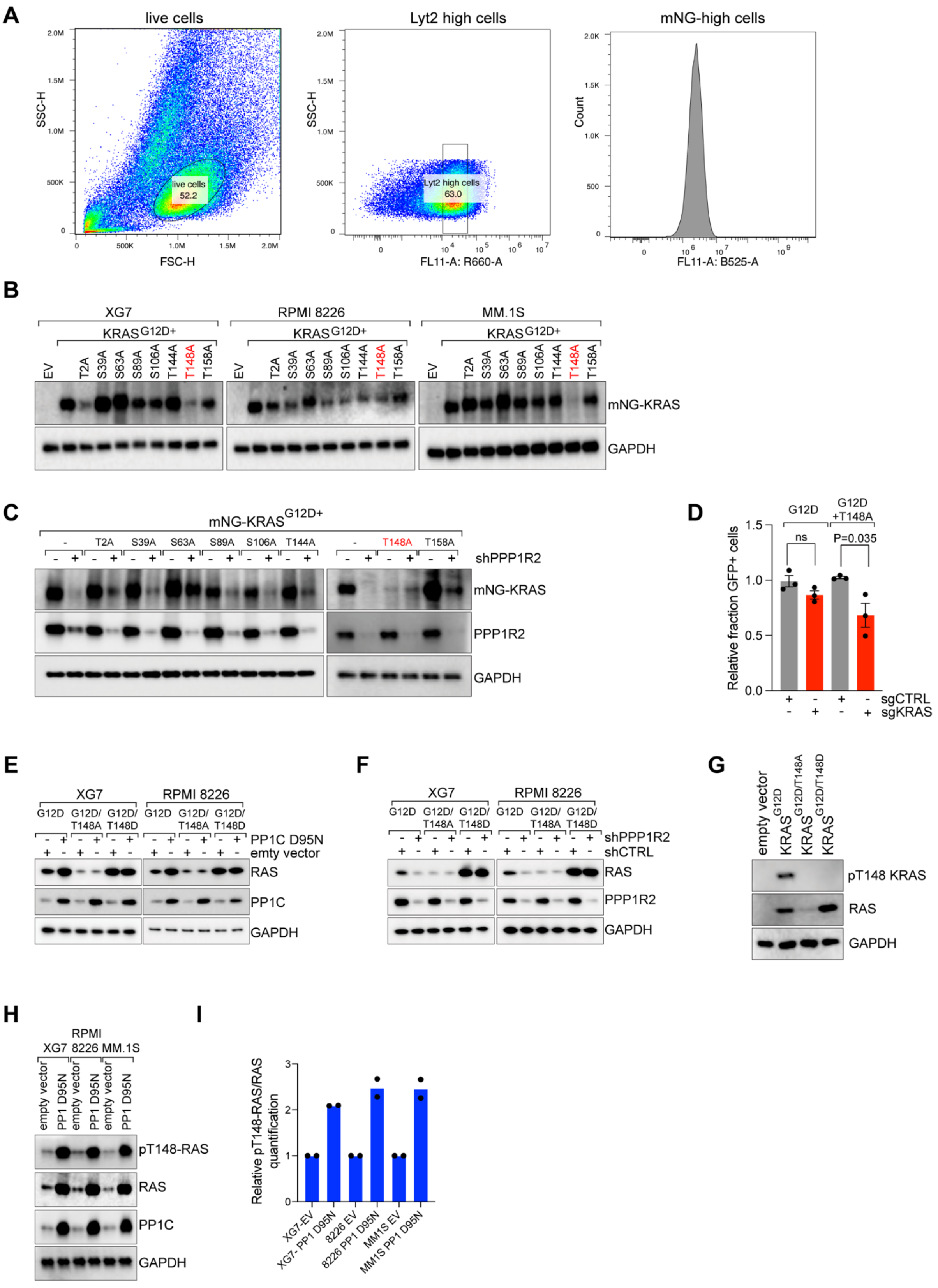
A
live cells
Lyt2 high cells
mNG-high cells
SSC-H
FSC-H
live cells 52.2
Lyt2 high cells 63.0
FL11-A: R660-A
Count
FL11-A: B525-A
B
XG7
RPMI 8226
MM.1S
KRAS G12D+
EV
T2A
S39A
S63A
S89A
S106A
T144A
T148A
T158A
mNG-KRAS
GAPDH
C
mNG-KRAS G12D+
shPPP1R2
PPP1R2
D
G12D
G12D +T148A
ns
P=0.035
Relative fraction GFP+ cells
sgCTRL
sgKRAS
E
G12D/ T148A
G12D/ T148D
PP1C D95N
emty vector
RAS
PP1C
F
shCTRL
G
empty vector
KRAS G12D
KRAS G12D/T148A
KRAS G12D/T148D
pT148 KRAS
H
empty vector
PP1 D95N
pT148-RAS
I
Relative pT148-RAS/RAS quantification
XG7-EV
XG7- PP1 D95N
8226 EV
8226 PP1 D95N
MM1S EV
MM1S PP1 D95N

Supplementary Figure 2. T148 of KRAS is directly targeted by PP1C. A) FACS analysis workflow to determine mNG-KRAS expression of various phospho-mutants. **B)** Immunoblot analysis of mNG-KRAS^{G12D} and GAPDH in XG7, RPMI 8226, and MM.1S cells harboring indicated mutations, n=3. **C)** Western blot analysis for mNG-KRAS, PPP1R2, and GAPDH in XG7 cells expressing indicated mNG-KRAS mutants and either shCTRL or shPPP1R2.1. **D)** Average normalized KRAS^{G12D} or KRAS$^{G12D+T148A}$ CRISPR-mediated rescue viability data following 12 days of KRAS knockout, n=3, error bars depict SEM. **E)** Western blot analysis of RAS, PP1C and GAPDH with empty vector or DN PP1C in XG7 and RPMI 8226 cells expressing KRAS^{G12D}, KRAS$^{G12D+T148A}$,or KRAS$^{G12D+T148D}$, n=2. **F)** Western blot analysis of RAS, PPP1R2 and GAPDH transduced with shCTRL or shPPP1R2.1 in XG7 and RPMI 8226 cells expressing KRAS^{G12D}, KRAS$^{G12D+T148A}$,or KRAS$^{G12D+T148D}$, n=2. **G)** Western blot analysis of pT148 KRAS, RAS and GAPDH in XG7 cells expressing empty vector, KRAS^{G12D}, KRAS$^{G12D+T148A}$,or KRAS$^{G12D+T148D}$, n=2. **H)** Western blot analysis of pT148 KRAS, RAS, PP1C and GAPDH in XG7, RPMI 8226 and MM.1S cells expressing empty vector, or DN PP1C, n=2 **I)** Ratio of pT148-RAS to RAS from quantified blots (from Figure S2H) in XG7, RPMI 8226, and MM.1S cells expressing empty vector or DN PP1C.

Figure S3

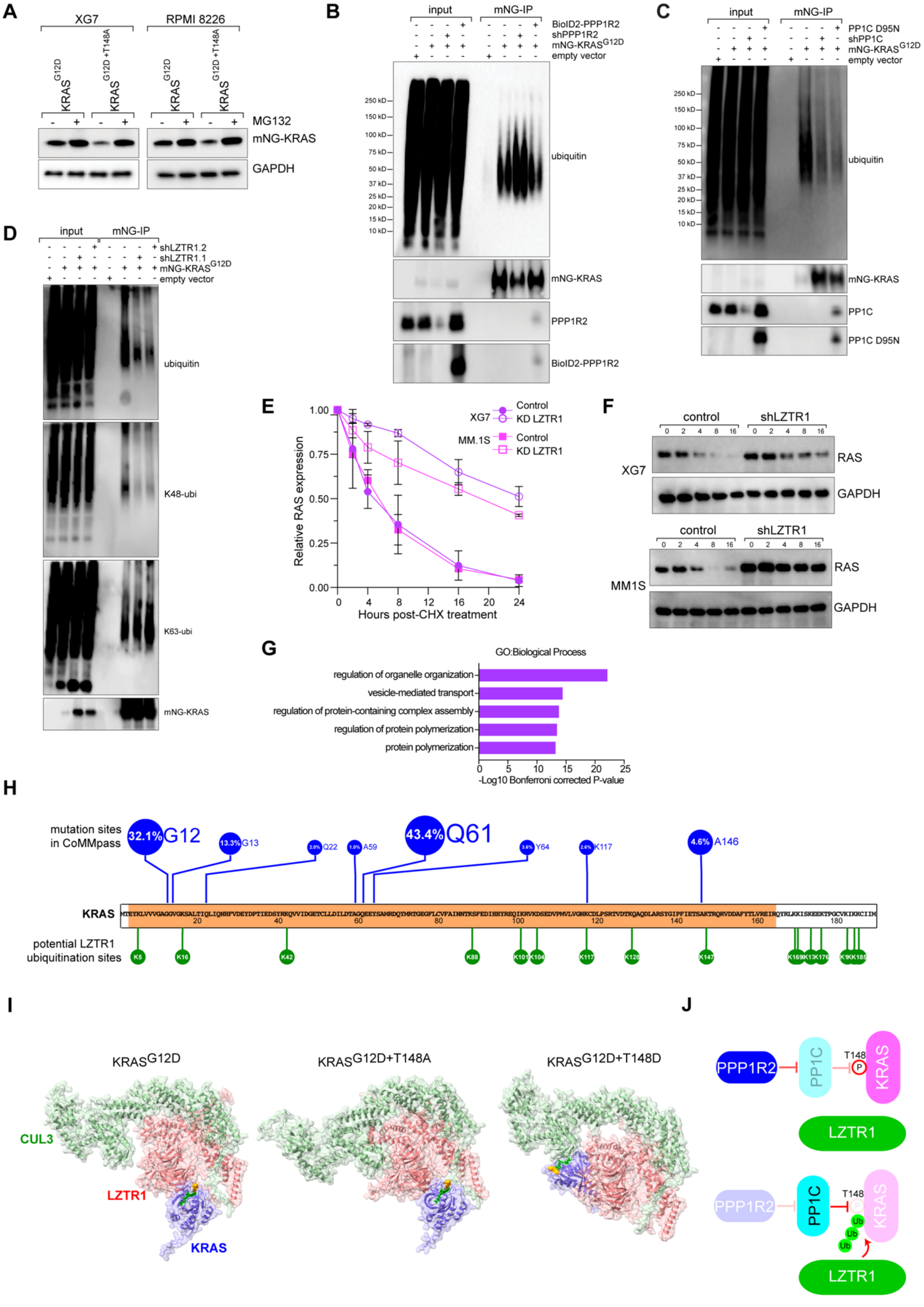

A
XG7
RPMI 8226
MG132
mNG-KRAS
GAPDH
B
input
mNG-IP
BioID2-PPP1R2
shPPP1R2
empty vector
ubiquitin
mNG-KRAS
PPP1R2
BioID2-PPP1R2
C
PP1C D95N
shPP1C
ubiquitin
PP1C
D
shLZTR1.2
shLZTR1.1
K48-ubi
K63-ubi
E
Relative RAS expression
Hours post-CHX treatment
Control
KD LZTR1
MM.1S
F
control
shLZTR1
RAS
MM1S
G
GO:Biological Process
regulation of organelle organization
vesicle-mediated transport
regulation of protein-containing complex assembly
regulation of protein polymerization
protein polymerization
-Log10 Bonferroni corrected P-value
H
mutation sites in CoMMpass
potential LZTR1 ubiquitination sites
KRAS
I
CUL3
LZTR1
J
PPP1R2
PP1C
T148

Supplementary Figure 3. PPP1R2 and PP1C regulate RAS ubiquitination modification. A) Western blot analysis of mNG-KRAS^{G12D}, mNG-KRAS$^{G12D+T148A}$,and GAPDH in XG7 and RPMI 8226 cells treated with or without 10 nM MG132 for 8 hours, n=3. **B)** Western blot analysis of ubiquitin binding following mNG-KRAS pulldown in cells transduced with empty vector, shPPP1R2.1, or BioID2-PPP1R2, n=2. **C)** Western blot analysis of ubiquitin binding following mNG-KRAS pulldown in cells transduced with empty vector, shPP1C, or DN PP1C, n=3. **D)** Western blot analysis of total, K48, and K63 ubiquitin binding following mNG-KRAS pulldown in cells transduced with empty vector or shLZTR1, n=2. **E)** Quantification of immunoblots of RAS expression normalized to GAPDH from XG7 and MM.1S with shCTRL or shLZTR1 following a time course of treatment with 10 nM cycloheximide (CHX) for the indicated timepoints (n=2; error bars depict standard deviation; representative blots in Fig. S3F). **F)** Representative western blots from panel E. G) Bar graph of Gene Ontology enrichment from LZTR1-BioID2 experiment. **H)** Schematic of KRAS oncogenic hotspot mutations and putative lysine ubiquitination sites. **I)** AlphaFold modeling of KRAS^{G12D} with additional T148A or T148D mutation to model a negative charge at this position. K147 is highlighted in yellow and GDP is in green. **J)** Model of T148-dependent regulation of RAS protein stability, in which phosphorylation at T148 protects KRAS from LZTR1-mediated degradation.

Figure S4

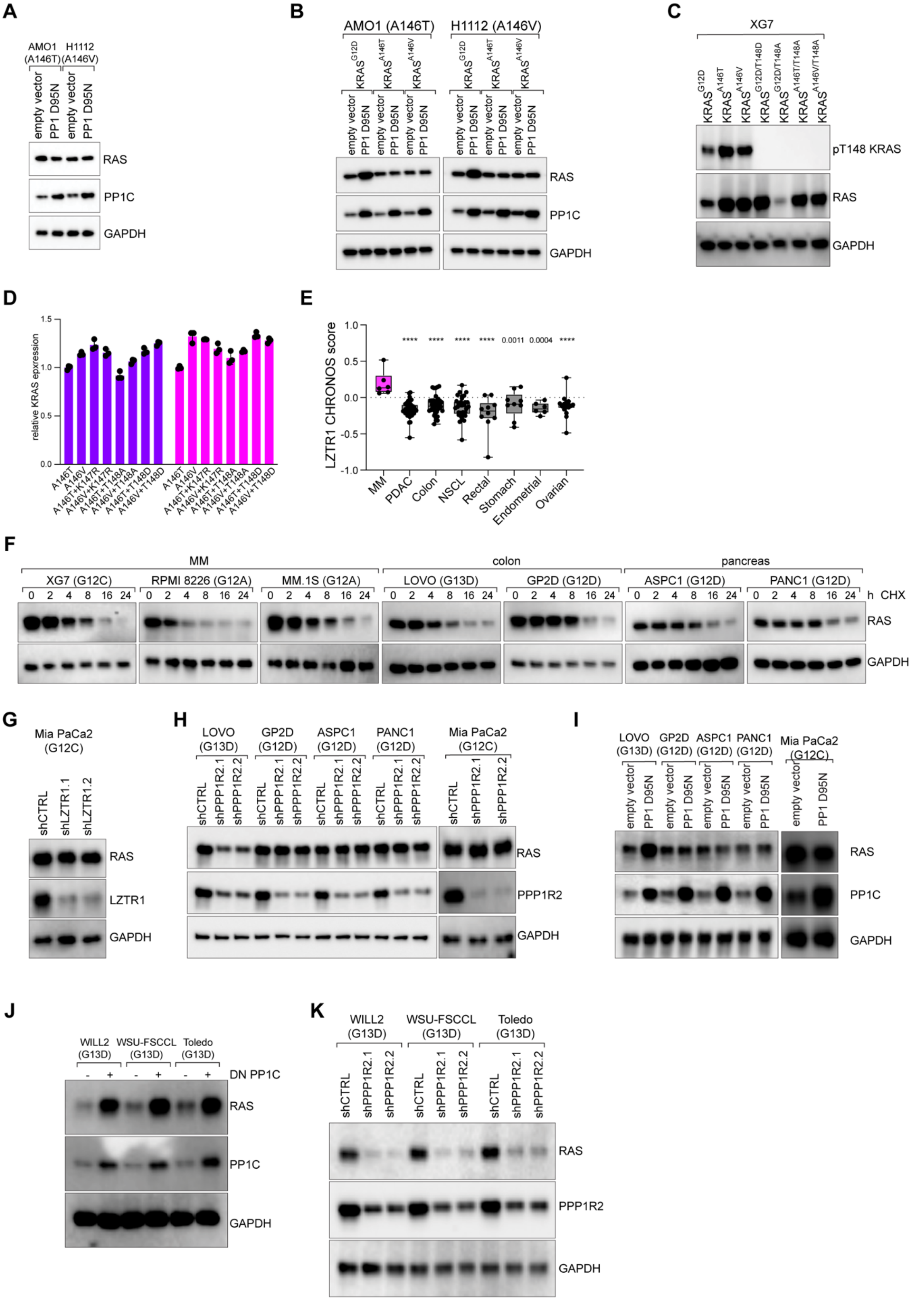

A
AMO1 (A146T)
H1112 (A146V)
empty vector
PP1 D95N
RAS
PP1C
GAPDH
B
AMO1 (A146T)
H1112 (A146V)
KRAS G12D
KRAS A146T
KRAS A146V
RAS
PP1C
GAPDH
C
XG7
pT148 KRAS
RAS
GAPDH
D
relative KRAS epxression
E
LZTR1 CHRONOS score
MM
PDAC
Colon
NSCL
Rectal
Stomach
Endometrial
Ovarian
0.0011
0.0004
F
MM
colon
pancreas
XG7 (G12C)
RPMI 8226 (G12A)
MM.1S (G12A)
LOVO (G13D)
GP2D (G12D)
ASPC1 (G12D)
PANC1 (G12D)
h CHX
RAS
GAPDH
G
Mia PaCa2 (G12C)
shCTRL
shLZTR1.1
shLZTR1.2
LZTR1
H
shPPP1R2.1
shPPP1R2.2
PPP1R2
I
J
WILL2 (G13D)
WSU-FSCCL (G13D)
Toledo (G13D)
DN PP1C
K

Supplementary Figure 4. A146 mutations protect KRAS from LZTR1. A) Western blot analysis of RAS, PP1C, and GAPDH in AMO1 and H1112 lines following expression of empty vector or DN PP1C, n=3. **B)** Western blot analysis of RAS, PP1C, and GAPDH after transducing empty vector or DN PP1C in AMO1 and H1112 cells expressing empty vector, KRAS^{G12D}, KRAS^{A146T},or KRAS^{A146V}, n=2. **C)** Western blot analysis of pT148 KRAS, RAS and GAPDH in XG7 cells expressing empty vector, KRAS^{G12D}, KRAS^{A146T}, KRAS^{A146V}, $\text{KRAS}^{A146T+T148A}$,or $\text{KRAS}^{A146V+T148A}$, n=2. **D)** Average normalized mNG-KRAS mutations expressing shCTRL or shLZTR.1 from FACS analysis, n=3, error bars depict standard deviation. **E)** Box plot of KRAS LZTR1 CHRONOS score from depmap for indicated tumor types, with MM highlighted in pink. **F)** Immunoblot analysis of RAS expression within indicated cell lines following treatment with 10 nM cycloheximide (CHX) for the indicated timepoints, n=2. **G)** Immunoblots of RAS, LZTR1, and GAPDH following expression of control or LZTR1 shRNAs in Mia PaCa2, n=2. **H-I)** Western blot analysis of RAS expression following transduction with control shRNA PPP1R2 (**H**) or following ectopic expression of DN PP1C **(I)**, n=3. **J-K**) Western blot analysis of RAS expression in WILL2, WSU-FSCCL, or Toledo GCB DLBCL cell lines following ectopic expression of DN PP1C (**J**), or following transduction with control shRNA or shRNAs targeting PPP1R2 (**K**), (n=3).

Figure S5

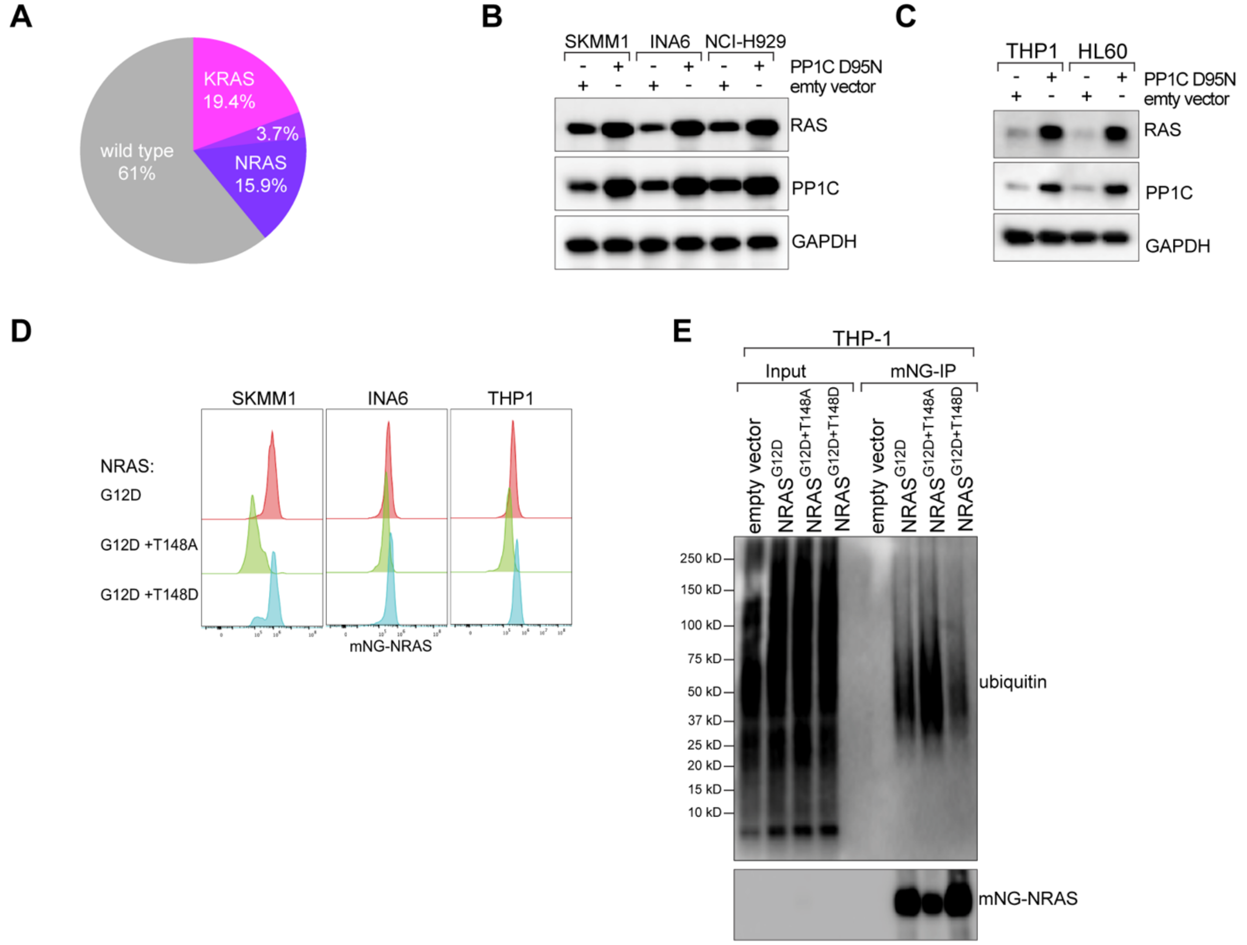

Supplementary Figure 5. PPP1R2 and PP1C control NRAS stability. A) Pie chart showing RAS mutation distribution in MM from MMRF CoMMpass data for tumors harboring KRAS (pink), NRAS (purple), both KRAS and NRAS (light purple), or wild-type RAS (gray). **B**) Western blot analysis of RAS, PP1C, and GAPDH with empty vector or DN PP1C in SKMM1, INA6, and NCI-H929 cells (n=3). **C**) Western blot analysis of RAS, PP1C, and GAPDH with empty vector or DN PP1C in THP1 and HL60 cells (n=3). **D**) Representative FACS data of mNG-KRAS^{G12D}, KRAS$^{G12D+T148A}$,or KRAS$^{G12D+T148D}$, n=2. **E)** Western blot analysis of ubiquitin binding following mNG-NRAS pulldown in cells transduced with indicated NRAS mutants in THP1 AML cells, n=3.

Figure S6

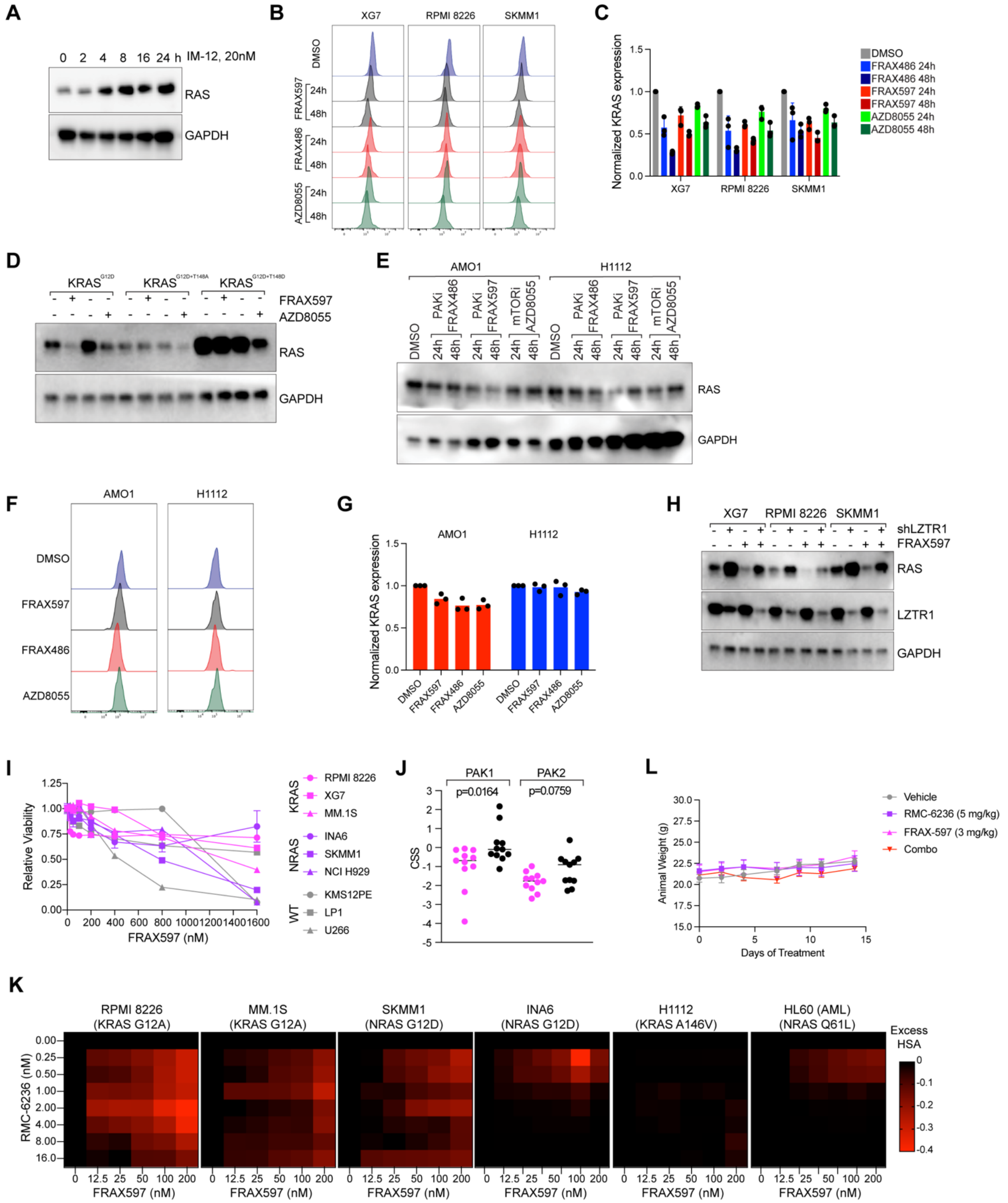

Supplementary Figure 6. PAK1/2 inhibition blocks T148 phosphorylation. A) Western blot analysis of RAS and GAPDH after treatment with GSK3B inhibitor, IM-12, at 20 nM for 2, 4, 8, 16, and 24 hours (n=2). **B**) Representative FACS data of mNG-KRASG12D after treatment with

PAKi (FRAX597 500 nM, FRAX486 500 nM, AZD8055 100 nM) for 24 and 48 hours (n=3).**C**) Quantification of FACS data from (B) normalized to DMSO for indicated drugs and cell lines (n=3; error bars depict standard deviation). **D)** Western blot analysis of RAS and GAPDH in XG7 cells expressing $KRAS^{G12D}$, $KRAS^{G12D+T148A}$,or $KRAS^{G12D+T148D}$ after treated with 500 nM FRAX597 or 100 nM AZD8055 for 24 h. **E)** Western blot analysis of RAS and GAPDH following treatment with FRAX597 500 nM, FRAX486 500 nM, and AZD8055 100 nM for 24 and 48 hours in AMO1 and H1112 cells. **F**) Representative FACS data of mNG-KRASG12D after treatment with PAKi (FRAX597 500 nM, FRAX486 500 nM, AZD8055 100 nM) for 48 hours in AMO1 and H1112 cells. **G**) Average normalized FACS data from mNG-KRASG12D with indicated inhibitor treatment from (E) in AMO1 and H1112 cells (n=3; error bars depict standard deviation). **H)** Western blot analysis of RAS, LZTR1, and GAPDH in XG7, RPMI 8226, and SKMM1 cells treated with 500 nM FRAX597 for 24 hours and expressing shCTRL or shLZTR1 (n=4).**I**) Cell viability following 4 days of treatment with FRAX597 at the indicated doses and in the indicated KRAS-dependent, NRAS-dependent, and RAS-independent MM lines, n=2. **J**) CRISPR Screen Scores (CSS) for PAK1 and PAK2 in RAS-dependent (pink) or RAS-independent (black) MM lines. Data adapted from (*13*). **K**) Excess HSA drug synergy matrices for indicated MM and AML lines treated with titrations of FRAX597 (x-axis) and RMC-6236 (y-axis). Cell viability was measured via CCK-8 at 4 days, n=3. **L**)Weights of mice from MM.1S xenograft experiment treated with indicated drugs.
